# Propofol abolishes torsade de pointes in different models of acquired long QT syndrome

**DOI:** 10.1038/s41598-020-69193-7

**Published:** 2020-07-22

**Authors:** Christian Ellermann, Hilke Könemann, Julian Wolfes, Benjamin Rath, Felix K. Wegner, Kevin Willy, Dirk G. Dechering, Florian Reinke, Lars Eckardt, Gerrit Frommeyer

**Affiliations:** 1 Department of Cardiology II (Electrophysiology), University Hospital Münster, Albert-Schweitzer-Campus 1, 48149 Münster, Germany; 2 Klinik für Kardiologie II – Rhythmologie, Universitätsklinikum Münster, Albert-Schweitzer Campus 1, 48149 Münster, Germany

**Keywords:** Cardiovascular biology, Experimental models of disease, Preclinical research, Translational research, Risk factors

## Abstract

There is conflicting evidence regarding the impact of propofol on cardiac repolarization and the risk of torsade de pointes (TdP). The purpose of this study was to elucidate the risk of propofol-induced TdP and to investigate the impact of propofol in drug-induced long QT syndrome. 35 rabbit hearts were perfused employing a Langendorff-setup. 10 hearts were perfused with increasing concentrations of propofol (50, 75, 100 µM). Propofol abbreviated action potential duration (APD_90_) in a concentration-dependent manner without altering spatial dispersion of repolarization (SDR). Consequently, no proarrhythmic effects of propofol were observed. In 12 further hearts, erythromycin was employed to induce prolongation of cardiac repolarization. Erythromycin led to an amplification of SDR and triggered 36 episodes of TdP. Additional infusion of propofol abbreviated repolarization and reduced SDR. No episodes of TdP were observed with propofol. Similarly, ondansetron prolonged cardiac repolarization in another 13 hearts. SDR was increased and 36 episodes of TdP occurred. With additional propofol infusion, repolarization was abbreviated, SDR reduced and triggered activity abolished. In this experimental whole-heart study, propofol abbreviated repolarization without triggering TdP. On the contrary, propofol reversed prolongation of repolarization caused by erythromycin or ondansetron, reduced SDR and thereby eliminated drug-induced TdP.

## Introduction

Up to 22% of patients treated on intensive care units experience ventricular arrhythmias and have a higher mortality compared to patients without heart rhythm disturbances^1^. Many risk factors have been identified over the past decades and include individual characteristics of each patient but also external factors such as use of pharmacological agents that impair repolarization reserve. Since propofol is commonly used in anaesthesia and intensive care medicine, the impact of propofol alone or in combination with other drugs that influence cardiac electrophysiology is of great interest. As a consequence, some studies have already investigated the electrophysiological effects of propofol in vivo and vitro:

Intravenous application of propofol has direct impact on several ion currents including I_Na_, some potassium channels (I_Ks_, I_K,to_, I_K,1_), and I_Ca,L_^2–5^. However, previous clinical and experimental studies report conflicting data regarding its impact on ventricular repolarization and potential proarrhythmic effects. Higashijima et al. described a significant abbreviation of QTc interval during anaesthetic induction mediated by propofol^6^. In contrast, a recent study demonstrated an increase in ventricular repolarization duration calculated by Fridericia-corrected QT interval with propofol^7^. Additionally, T_peak_ to T_end_ interval (T_peak_-T_end_) was significantly amplified in the presence of propofol in this study. An increased T_peak_-T_end_ interval is a surrogate for an amplified transmural dispersion of repolarization which in turn represents a major risk factor for drug-induced arrhythmias^8^. In contrast to this study, no significant changes of QTc or T_peak_-T_end_ have been reported during propofol infusion in children^9^.

Some other experimental studies have investigated effects of propofol in different models of long QT syndrome (LQTS): In healthy and transgenic LQT2 and LQT3 rabbits, propofol administration resulted in an increase of QT index resulting in arrhythmia-related death in two LQT2 rabbits^5^. In another study employing a model of (drug-induced) LQTS, propofol reduced the action potential duration increase mediated by erythromycin^10^.

In conclusion, existing studies report conflicting data concerning propofol-induced changes in repolarization duration and heterogeneity and provocation of arrhythmias. Therefore, the purpose of the present study was to elucidate propofol’s impact in a sensitive model of repolarization disorders. Previous experimental studies have solely investigated the effect of propofol infusion on ventricular repolarization duration and other ECG markers (e.g. T_peak_-T_end_) but did not investigate other proarrhythmic mechanisms such as dispersion of repolarization or action potential shape. Thus, this study aimed at elucidating further potential mechanisms in arrhythmia initiation induced by propofol.

## Methods

All experimental protocols were approved by the local animal care committee (Landesamt für Natur, Umwelt und Verbraucherschutz Nordrhein-Westfalen, Germany) and were carried out in accordance with the Guide for the Care and Use of Laboratory Animals published by the US National Institutes of Health (NIH Publication No. 852-3, revised 1996). Since hearts served as their own control, no randomization of the hearts was performed.

The experimental setting of the antegradely-perfused Langendorff-heart has been described earlier extensively^11^. In short, 35 hearts of female New Zealand white rabbits were explanted and mounted to a Langendorff apparatus. Spontaneously beating hearts were perfused by a warmed, oxygenated (95% O_2_, 5% CO_2_) modified Krebs–Henseleit buffer (NaCl 118 mM, NaHCO_3_ 24.88 mM, d-glucose 5.55 mM, KCl 4.70 mM, Na-pyruvate 2 mM, CaCl_2_ 1.80 mM, KH_2_PO_4_ 1.18 mM, MgSO_4_ 0.83 mM) at a constant flow (52 mL/min) with a pressure around 90 mmHg. Monophasic action potentials (MAP) were acquired by eight specifically designed MAP catheters that were placed endo- and epicardially. Hearts were immersed in a warmed tissue bath, thereby enabling recording of a volume-conducted 12-lead ECG. Spontaneously beating hearts were mechanically AV node-ablated using surgical tweezers in order to perform the following stimulation protocol.

**Figure 1:**
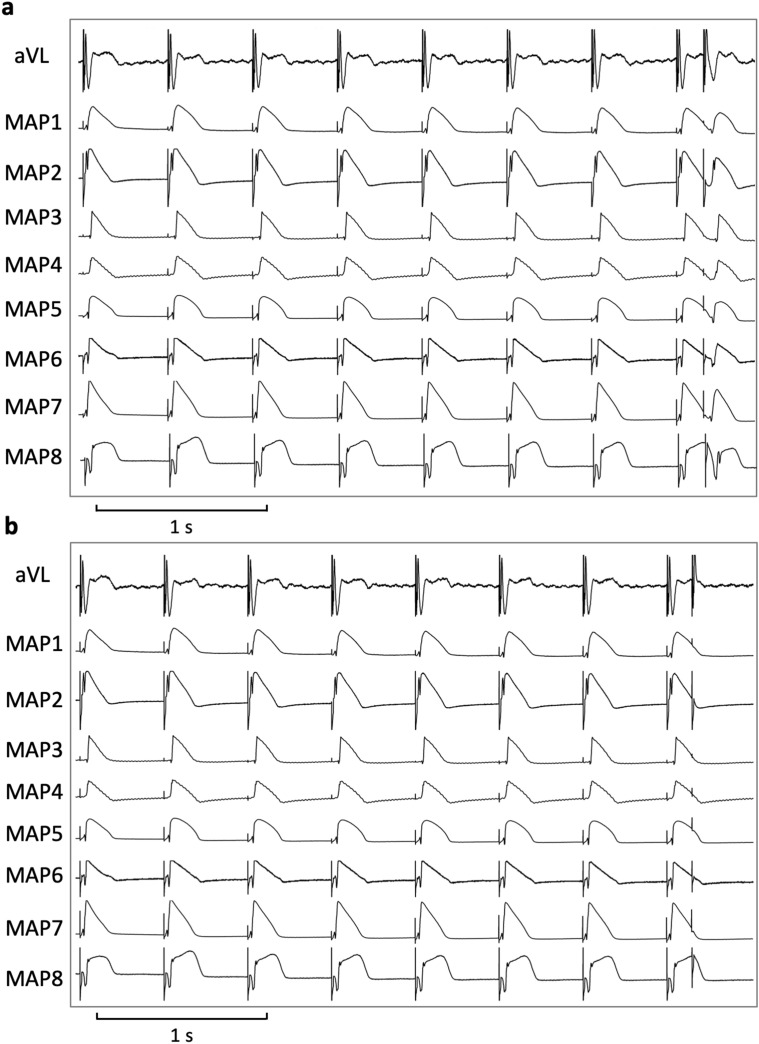
Determination of effective refractory periods (MAP = monophasic action potential).

Hearts were stimulated at seven different cycle lengths (900–300 ms), thus obtaining cycle-lengths dependent QT interval and action potential duration (APD_90_). APD_90_ was measured between the fastest upstroke and 90% of repolarization. Premature extra-stimuli (S_2_ and S_3_) were delivered to the hearts in order to assess ventricular vulnerability and to determine effective refractory periods (ERP) at different basic cycle-lengths (900–300 ms, see Fig. 1). In case sustained ventricular arrhythmias occurred after short-coupled extra-stimuli, hearts were defibrillated, and the pacing protocol was halted for 5 min to assure recovery of the hearts. Post-repolarization refractoriness (PRR) was calculated as the difference between ERP and APD_90_. Spatial dispersion of repolarization was determined by the difference of maximum and minimum of the APD_90_ of the eight MAPs. Configuration of action potentials is displayed by the ratio of APD_90_/APD_50_^12,13^.

Hearts were divided up into three different groups: In the first group, propofol in ascending concentrations (50, 75, 100 µM) was infused after generating baseline data and the protocol was repeated for each concentration. In this experimental arm, premature extra-stimuli were solely delivered after pacing the hearts at a basic cycle-length of 500 ms in order to abridge the experimental protocol. The second group was perfused with 300 µM erythromycin after generating baseline data. Afterwards, hearts were additionally treated with 75 µM propofol. The last group was infused with 5 µM ondansetron and thereafter 75 µM propofol was added. Before continuing the experimental protocol with a new drug or concentration, hearts were equilibrated for 15 min.

### Statistics

Electrograms and action potentials were recorded on a multi-channel recorder and digitalized at a rate of 1 kHz with a 12-bit resolution. Variables are shown as mean ± standard deviation. Statistical analyses were performed using SPSS Statistics for Windows (version 24.0). Drug effects on APD_90_, QT interval, dispersion of repolarization, action potential configuration (APD_90_/APD_50_), ERP and PRR were analysed employing Wilcoxon signed rank test. P values < 0.05 were considered to be statistically significant. Data are expressed as mean ± standard deviation.

## Results

### Propofol effects on ventricular repolarization and arrhythmia induction

**Figure 2:**
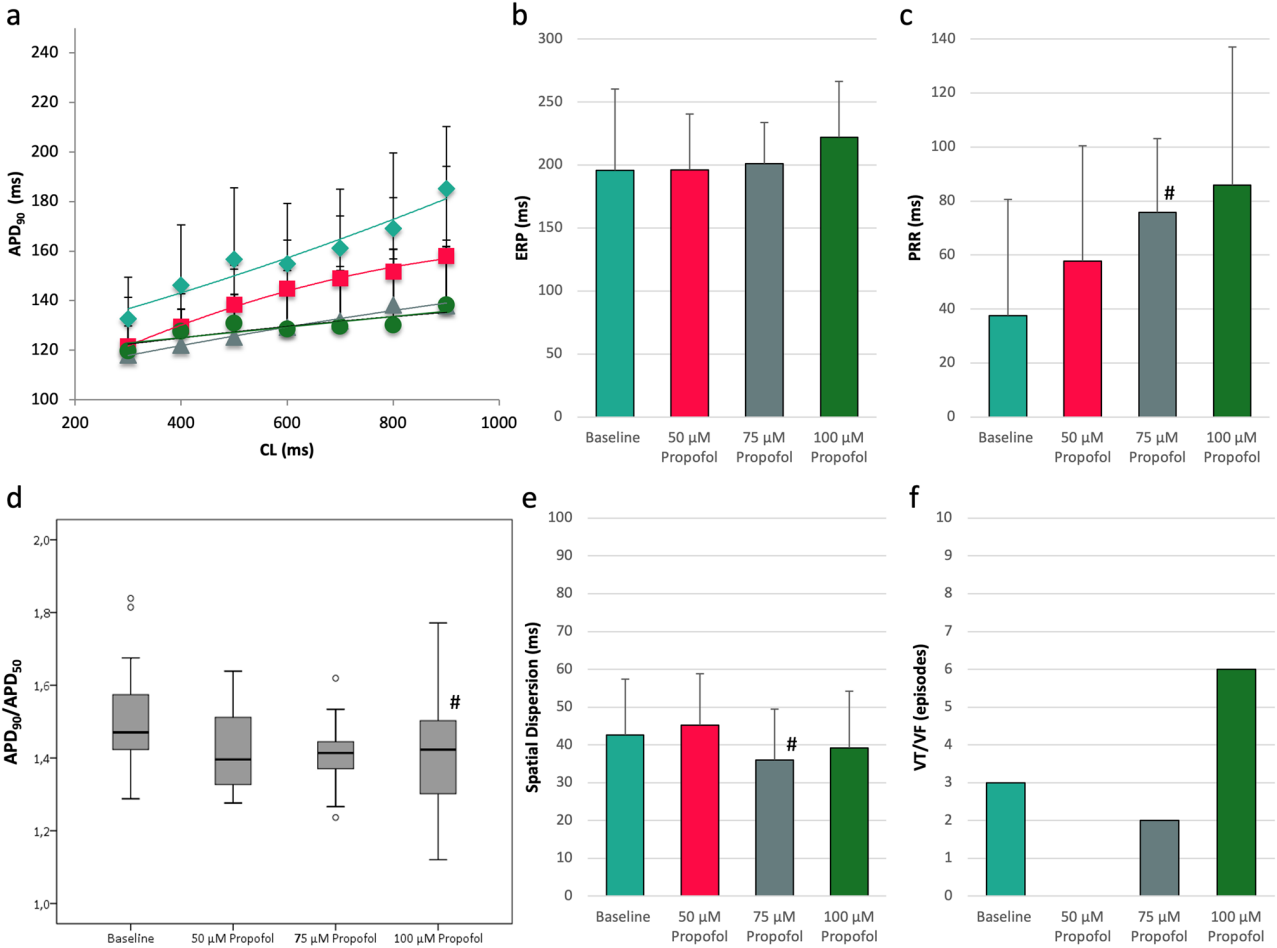
(**a**) Cycle-length dependent APD_90_ under baseline conditions (filled rhombus) and after treatment with 50 µM (filled square), 75 µM (filled traingle) or 100 µM (filled circle) propofol. (**b**) Impact of propofol on effective refractory periods (ERP). (**c**) Concentration-dependent effect of propofol on post-repolarization refractoriness (^#^p < 0.05 compared to baseline conditions). (**d**) Box plots of the ratio of action potential duration at 90% of repolarization (APD_90_) and action potential duration at 50% of repolarization (APD_50_). A decrease in APD_90_/APD_50_ represents a rectangulation of action potential. (**e**) Influence of propofol treatment on repolarization heterogeneity as indicated by spatial dispersion of repolarization (^#^p < 0.05 compared to baseline conditions). (**f**) Occurrence of ventricular fibrillation (VF) tachycardia (VT) induced by programmed ventricular fibrillation.

Infusion of propofol abbreviated APD_90_ in a concentration-dependent manner (baseline: 158 ± 25 ms; 50 µM: 142 ± 21 ms, p < 0.01 compared to baseline; 75 µM: 129 ± 19 ms, p < 0.01 compared to baseline; 100 µM: 129 ± 23 ms, p < 0.01 compared to baseline; Fig. 2) while QT interval remained relatively stable at the lowest propofol concentration and was slightly abbreviated under the influence of 75 and 100 µM propofol (baseline: 242 ± 30 ms; 50 µM: 243 ± 35 ms, p = ns; 75 µM: 241 ± 32 ms, p = 0.07; 100 µM: 239 ± 27 ms, p < 0.01; each p compared to baseline).

ERP were increased after propofol treatment in a concentration-dependent manner (baseline: 196 ± 65 ms; 50 µM: 196 ± 44 ms, p = ns; 75 µM: 201 ± 33 ms, p = ns; 100 µM: 222 ± 44 ms, p = ns), resulting in a significant amplification of PRR (baseline: 38 ± 43 ms; 50 µM: 58 ± 43 ms, p = ns; 75 µM: 76 ± 27 ms, p < 0.02; 100 µM: 86 ± 51 ms, p = ns).

Spatial dispersion of repolarization was not significantly altered at lowest or highest propofol concentration but was decreased with 75 µM propofol (baseline: 43 ± 15 ms; 50 µM: 45 ± 14 ms, p = ns; 75 µM: 36 ± 13 ms, p < 0.05; 100 µM: 39 ± 15 ms, p = ns).

The action potential shape which can be expressed by the ratio of APD_90_/APD_50_ was not significantly changed in the presence of 50 or 75 µM propofol (baseline: 1.48 ± 0.13, 50 µM: 1.44 ± 0.11, p = ns; 75 µM: 1.47 ± 0.11, p = ns). However, with the highest concentration tested (100 µM), a further rectangulation of action potential shape could be observed (1.42 ± 0.14, p = 0.01).

3 episodes of ventricular tachycardia or fibrillation were inducible by programmed ventricular stimulation (S_2_ and S_3_) under baseline conditions. No episodes occurred with 50 µM propofol (p = ns) while 2 episodes of VT/VF were inducible under the influence of 75 µM propofol (p = ns). With the highest propofol concentration (100 µM) 6 episodes of VT/VF were inducible (p = ns).

### Erythromycin

**Figure 3:**
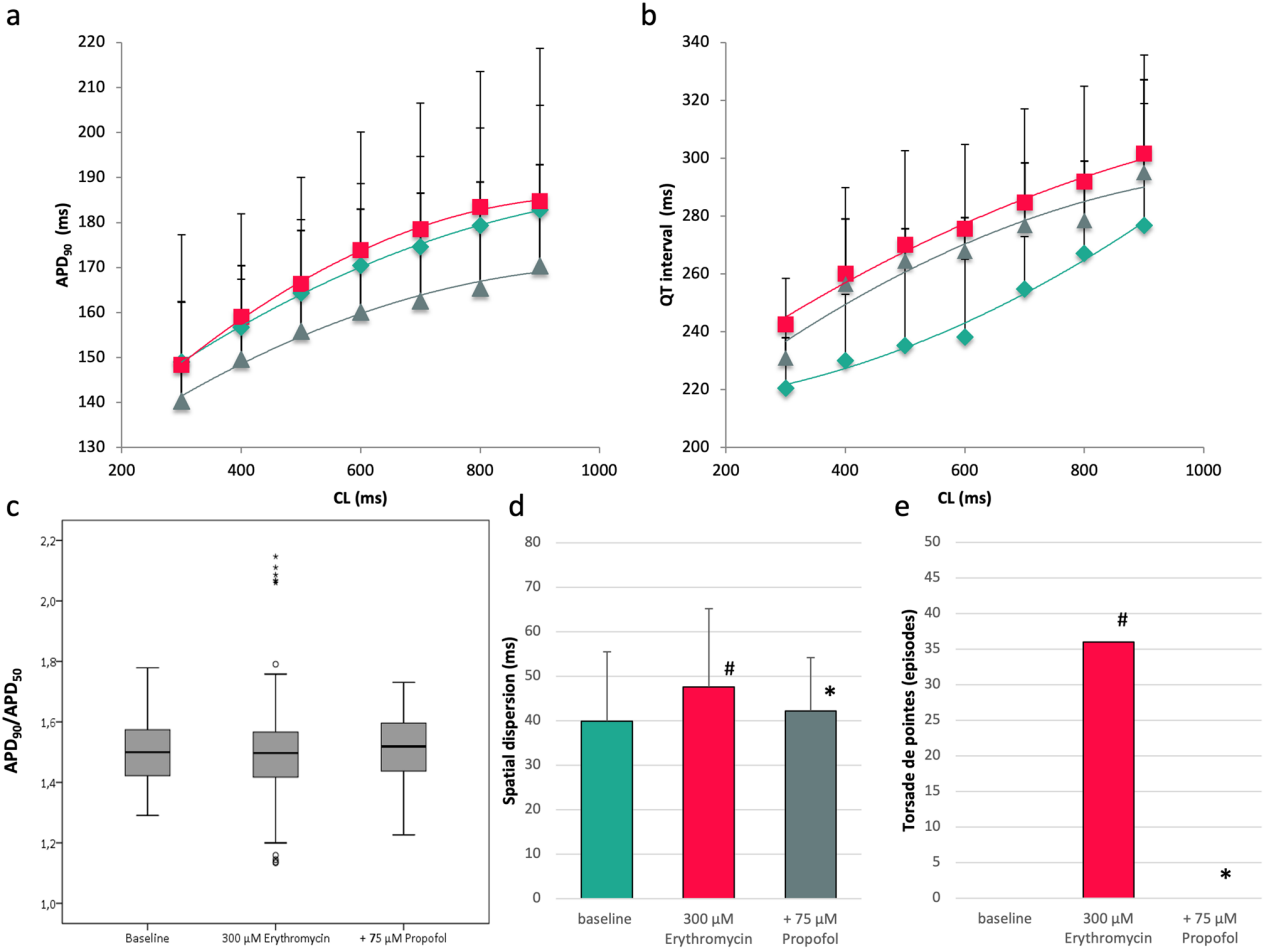
(**a**,**b**) Cycle-length dependent APD_90_ and QT interval under baseline conditions (filled rhombus), after treatment with 300 µM erythromycin (filled square) and after additional infusion of 75 µM propofol (filled triangle). (**c**) Box plots of the ratio of APD_90_ to APD_50_. (**d**) Impact of erythromycin and propofol on spatial dispersion of repolarization (^#^p < 0.05 compared to baseline conditions; *p < 0.05 compared to sole erythromycin infusion). (**e**) Occurrence of torsade de pointes under baseline conditions, with erythromycin and with the combination of erythromycin and propofol (^#^p < 0.05 compared to baseline conditions; *p < 0.05 compared to sole erythromycin treatment).

Treatment with 300 µM erythromycin prolonged QT interval from 246 ± 27 ms to 275 ± 30 ms (p < 0.01; Fig. 3) while APD_90_ was just slightly increased from 168 ± 18 ms to 171 ± 28 ms (p = ns). Propofol reversed these effects and abbreviated QT interval to 267 ± 18 ms (p < 0.01) and APD_90_ to 158 ± 23 ms (p < 0.01). Spatial dispersion of repolarization was significantly amplified in the presence of erythromycin (baseline: 40 ± 16 ms; erythromycin: 48 ± 18 ms, p < 0.01) and reduced by the additional treatment with propofol to 42 ± 12 ms (p = 0.01 compared to erythromycin).

There was a trend towards an increase of APD_90_/APD_50_ after infusion of erythromycin from 1.49 ± 0.12 to 1.52 ± 0.19 (p = 0.13), representing a triangulation of action potential shape. With propofol, a non-significant decrease of the APD_90_/APD_50_ ratio was observed (1.48 ± 0.10, p = 0.21).

No torsade de pointes occurred under baseline conditions. However, 36 episodes of torsade de pointes spontaneously occurred after treatment with erythromycin (p < 0.05 compared to baseline) and were completely eliminated in the presence of propofol (0 episodes, p < 0.05).

### Ondansetron

**Figure 4:**
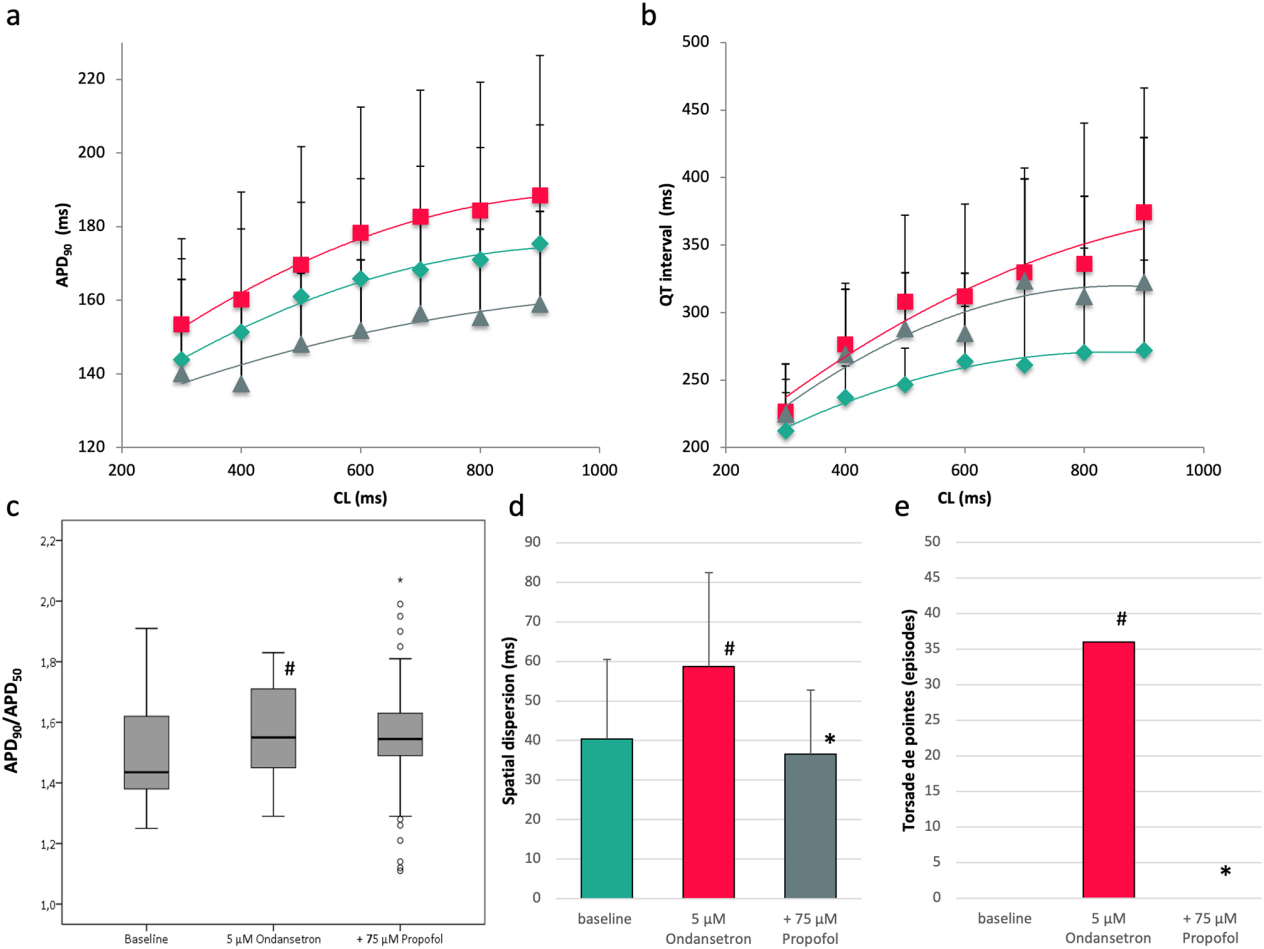
(**a**,**b**) Cycle-length dependent APD_90_ and QT interval under baseline conditions (filled rhombus), after treatment with 5 µM ondansetron (filled square) and after additional infusion of 75 µM propofol (filled triangle). (**c**) Box plots of the ratio of APD_90_ to APD_50_ (^#^p < 0.05 compared to baseline conditions) (**d**) Influence of ondansetron and propofol on spatial dispersion of repolarization (^#^p < 0.05 compared to baseline conditions; *p < 0.05 compared to sole ondansetron administration). (**e**) Occurrence of torsade de pointes under baseline conditions, with ondansetron and with the combination of ondansetron and propofol (^#^p < 0.05 compared to baseline conditions; *p < 0.05 compared to sole ondansetron treatment).

With ondansetron, an increase of QT interval from 252 ± 46 ms to 309 ± 68 ms (p = ns; Fig. 4) and of APD_90_ from 162 ± 28 to 174 ± 32 ms (p < 0.01) was observed. Additional infusion of propofol abbreviated QT interval (to 289 ± 61 ms, p = ns) as well as APD_90_ (to 150 ± 23 ms, p < 0.01). Spatial dispersion of repolarization was amplified after ondansetron infusion (from 40 ± 20 ms to 59 ± 24 ms, p < 0.01) and substantially reduced under the additional influence of propofol (37 ± 16 ms, p < 0.01).

The APD_90_/APD_50_ ratio was significantly increased in the presence of ondansetron from 1.53 ± 0.18 to 1.56 ± 0.15 (p < 0.05). Propofol did not significantly alter the APD_90_/APD_50_ ratio (1.53 ± 0.19; p = ns).

**Figure 5:**
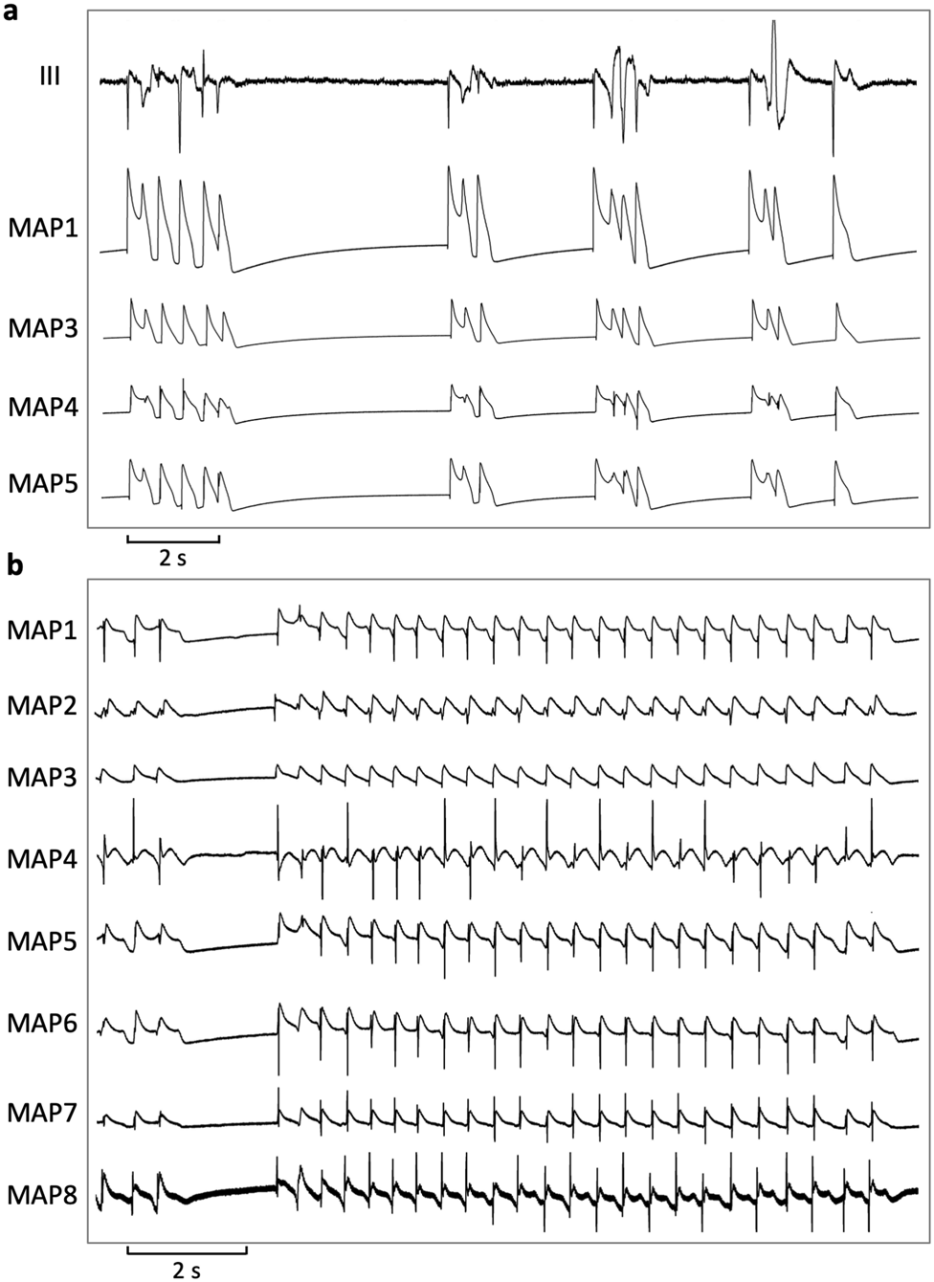
(**a**) Representative example of early afterdepolarizations induced by erythromycin (MAP = monophasic action potential). (**b**) Spontaneously occurring polymorphic ventricular tachycardia resembling torsade de pointes after ondansetron treatment.

No episodes of torsade de pointes occurred in the spontaneously beating, AV-blocked hearts under baseline conditions. With ondansetron, 36 episodes of torsade de pointes were observed (p < 0.02, Fig. 5). Again, propofol treatment eliminated torsade de pointes in each heart (0 episodes, p < 0.02).

## Discussion

To our best knowledge, this is the first experimental whole-heart study investigating propofol’s effects on cardiac electrophysiology in different models of acquired long QT syndrome. This study demonstrates that sole propofol infusion slightly abbreviates ventricular repolarization without triggering torsade de pointes. Furthermore, administration of propofol on top of proarrhythmic agents such as erythromycin or ondansetron reduces repolarization and spatial dispersion of repolarization and thereby eliminates torsade de pointes.

### Impact of propofol on cardiac electrophysiology

In the present study, propofol induced a significant abbreviation of cardiac repolarization as indicated by APD_90_ and QT interval. This is in line with the majority of former clinical studies investigating repolarization duration under the influence of propofol^14^. Previous data concerning propofol’s influence on T_peak_-T_end_ is equivocal. While a recent clinical study showed a prolonged T_peak_-T_end_ interval with propofol^7^, no changes were observed in another trial with a paediatric study cohort^9^. T_peak_-T_end_ interval has been proposed as a surrogate for transmural dispersion of cardiac repolarization^15^. In contrast to the QT interval that mediocrely predicts occurrence of torsade de pointes, an increased transmural dispersion of repolarization is a good indicator for drug-induced arrhythmias^8,13^. This study clearly indicates a stable dispersion of repolarization during propofol-treatment even at supratherapeutic concentrations. A stable dispersion of repolarization (even in the presence of a prolonged cardiac repolarization) is linked to a safe electrophysiologic profile of several antiarrhythmic drugs^16^.

Furthermore, the shape of action potential duration was transformed by the highest concentration of propofol to a more rectangular shape as indicated by a decrease in APD_90_/APD_50_. A rectangulation of action potential reduces the risk of arrhythmias and is mediated by an acceleration of phase 3 repolarization which reduces the time in the window voltage for calcium channel reactivation and subsequent triggered activity^12^. Thus, no arrhythmias were observed in bradycardic hearts even with the highest concentration of propofol used. As a consequence, this study highlights a good safety profile of propofol. With propofol, post-repolarization refractoriness was significantly lengthened. Prolongation of PRR protects the myocardium against premature beats, is therefore antiarrhythmic^11,17^ and a common pharmacological property of class I antiarrhythmic drugs. Consequently, ventricular vulnerability as tested by programmed ventricular stimulation was not increased with propofol.

In this study, supratherapeutic concentrations of propofol have been employed to determine adverse drug effects. Mean propofol concentration during anesthesia induction is 11.7 (± 5.0) µg/mL which equals approximately 65.6 µM^18^. However, since genetic polymorphisms in hepatic metabolizing enzymes (e.g. CYP2C9) may further increase propofol concentrations during anaesthesia^18^, higher plasma concentrations might be achieved. Therefore, concentrations of up to 100 µM have been employed in this study.

### Models of acquired long QT syndrome

With erythromycin, a marked prolongation of repolarization duration, an amplification of spatial dispersion of repolarization and a trend towards a triangulation of action potential shape were observed. This is in line with previous studies in which the I_Kr_ inhibitor erythromycin was employed to simulate LQT2 syndrome^11^. Similar results have been achieved for ondansetron which also inhibits hERG (human Ether-a-go-go Related Gene) potassium channels^19^. Accordingly, ondansetron augmented repolarization duration and amplified spatial dispersion of repolarization^20^.

**Figure 6:**
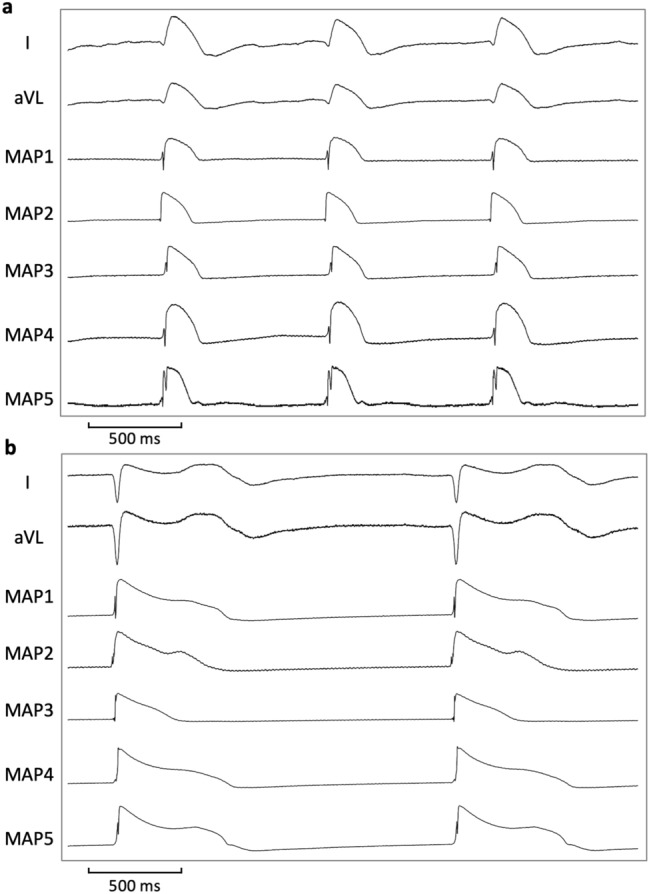
Illustrative example of action potential and ECG tracings under baseline conditions (**a**) and after administration of erythromycin (**b**) in spontaneously beating bradycardic hearts under hypokalemic conditions. With erythromycin, action potentials are substantially prolonged and triangulated. Of note, spatial dispersion of repolarization (as determined by the duration differences between MAP 5 and MAP 3 in (**b**)) is amplified. (MAP = monophasic action potential).

Erythromycin and ondansetron changed the shape of the action potential to a more triangular shape which can be explained by an inhibition of I_Kr_ (Fig. 6). This leads to a slowing of phase 3 repolarization which in turn prolongs the time frame in which early afterdepolarizations and subsequent torsade de pointes can be generated^12^. Consequently, early afterdepolarizations and torsade de pointes were observed with both drugs.

In contrast, infusion of propofol reversed the changes induced by erythromycin or ondansetron. To be more precise, propofol abbreviated repolarization and reduced spatial dispersion of repolarization in both groups. Previous studies demonstrated that a decrease of spatial dispersion of repolarization is a crucial antiarrhythmic mechanism in acquired long QT syndrome^11,17^. Recently, Bossu and colleagues^21^ elegantly demonstrated that reduction of spatial dispersion of repolarization induced by the I_Na,L_ inhibitor GS967 predominantly inhibits perpetuation of torsade de pointes in the chronic atrioventricular block dog. It is noteworthy that early afterdepolarization which are regarded as initiating mechanism were just slightly suppressed. Consequently, the prevention of perpetuation instead of prevention of the initiation of the arrhythmia can be regarded as the antiarrhythmic mechanism of GS967 in this study^21^. Similarly, the crucial antiarrhythmic action of propofol in this study might not be inhibition of triggered activity but rather prevention of perpetuation of torsade de pointes tachycardia.

There was a non-significant trend towards a rectangular action potential shape with additional propofol treatment. As a consequence, triggered activity (early afterdepolarizations or torsade de pointes) occurred neither in the erythromycin nor in the ondansetron group after additional propofol treatment. These results are in line with a previous study in which propofol reversed the prolongation of repolarization induced by erythromycin. However, no further mechanistic investigations were performed, and no arrhythmias were recorded due to the experimental setup^10^. These findings were confirmed in a clinical setting in which propofol reversed QT interval prolongation induced by sevoflurane^22^.

Surprisingly, in transgenic LQT2 rabbits, propofol prolonged repolarization and subsequently triggered torsade de pointes^5^. Even though above-mentioned studies indicated different effects of propofol in LQTS-linked arrhythmias, one would actually expect similar results since inhibition of I_Kr_ either by erythromycin or ondansetron is likely to result in similar electrophysiologic effects as observed in LQT2.

Of note, the electrophysiologic effects of propofol in acquired LQTS observed in this study are comparable to those obtained for the sodium current inhibitor mexiletine^17^.

## Limitations

The present study was conducted in isolated rabbit hearts. Therefore, a direct extrapolation to humans is not possible. However, previous studies indicate that the rabbit heart is a reasonable model for studying cardiac ion channel function and especially for investigating cardiac repolarization disorders^23^. Furthermore, the rabbit heart is particularly suitable for studying complex ventricular arrhythmias like ventricular fibrillation due to its effective size, which relates the size of the heart to the wavelength of the arrhythmia^24^. Following this concept as proposed by Panfilov^24^, the effective size of the rabbit heart is similar to the human heart leading to a similar arrhythmia pattern in both species.

However, this model does not allow precise statements concerning direct effects on ion channels. Reduction of repolarization duration induced by propofol can probably be explained by the predominant inhibition of sodium and calcium channels that overrides the effects of potassium channel block. Accordingly, distinct effects of propofol on different human cardiac channels have been described and this multi-channel inhibition most likely explains the results observed in this study: To be more precise, previous patch clamp studies reported that propofol inhibits human L-type calcium currents^25^, human sodium^3^ as well as human potassium channels^26^.

## Conclusion

The present study demonstrates a safe electrophysiologic profile of propofol even at high concentrations. Propofol abbreviated cardiac repolarization and did not bear the risk of proarrhythmia. Quite the contrary, propofol abbreviated repolarization duration in different models of acquired long QT syndrome, reduced spatial dispersion of repolarization and thereby eliminated drug-induced torsade de pointes. As a consequence, propofol might even be beneficial in drug-induced QT prolongation by reducing the risk of torsade de pointes.

## Data Availability

The datasets generated during and analysed during the current study are available from the corresponding author on reasonable request.
